# Digital health in the era of COVID-19: Reshaping the next generation of healthcare

**DOI:** 10.3389/fpubh.2023.942703

**Published:** 2023-02-15

**Authors:** Emnet Getachew, Tsegaye Adebeta, Seke G. Y. Muzazu, Loveness Charlie, Bibie Said, Hanna Amanuel Tesfahunei, Catherine Lydiah Wanjiru, Joan Acam, Violet Dismas Kajogoo, Samrawit Solomon, Mary Gorret Atim, Tsegahun Manyazewal

**Affiliations:** ^1^Center for Innovative Drug Development and Therapeutic Trials for Africa (CDT-Africa), College of Health Sciences, Addis Ababa University, Addis Ababa, Ethiopia; ^2^Department of Public Health, College of Health Science, Arsi University, Asella, Ethiopia; ^3^Outpatient Department, Ethiopian Airlines Medical Unit, Addis Ababa, Ethiopia; ^4^Enteric Diseases and Vaccines Research Unit, Centre for Infectious Disease Research in Zambia (CIDRZ), Lusaka, Zambia; ^5^KNCV TB Foundation, Challenge TB Project, Blantyre, Malawi; ^6^Outpatient Department, Kibong'oto National Tuberculosis Hospital, Moshi, Kilimanjaro, Tanzania; ^7^Department of Public Health, Hager Biomedical Research Institute, Asmara, Eritrea; ^8^Outpatient Department, Pope John's Hospital Aber, Atapara, Uganda; ^9^School of Public Health, Saint Paul's Hospital Millennium Medical College, Addis Ababa, Ethiopia; ^10^Soroti Regional Referral Hospital, Soroti, Uganda

**Keywords:** digital health, technology, DHIs, COVID-19, pandemic

## Abstract

COVID-19 is one of the most deadly diseases to have stricken us in recent decades. In the fight against this disease, governments and stakeholders require all the assistance they can get from various systems, including digital health interventions. Digital health technologies are supporting the tracking of the COVID-19 outbreak, diagnosing patients, expediting the process of finding potential medicines and vaccines, and disinfecting the environment, The establishment of electronic medical and health records, computerized clinical decision support systems, telemedicine, and mobile health have shown the potential to strengthen the healthcare system. Recently, these technologies have aided the health sector in a variety of ways, including prevention, early diagnosis, treatment adherence, medication safety, care coordination, documentation, data management, outbreak tracking, and pandemic surveillance. On the other hand, implementation of such technologies has questions of cost, compatibility with existing systems, disruption in patient-provider interactions, and sustainability, calling for more evidence on clinical utility and economic evaluations to help shape the next generation of healthcare. This paper argues how digital health interventions assist in the fight against COVID-19 and their opportunities, implications, and limitations.

## Introduction

The World Health Organization has suggested that countries keep maximizing the opportunities for digital health interventions (DHIs) to accelerate sustainable health development and universal health coverage. DHIs are applications of smartphones, health information technology, wearable devices, telemedicine, and personalized medicine to facilitate healthcare and attain intended health outcomes (1, 2). They enhance patient care by facilitating treatment adherence and monitoring, person-centered care, laboratory diagnosis, data management, disease surveillance, drug-safety monitoring, and professional development (3–5). Healthcare institutions are looking for digital health innovations to improve care quality by integrating various technologies. However, there are impediments that countries, especially those with limited resources, face when implementing digital health, calling for a good grasp of how to develop appropriate strategies to benefit the most of digital health-enabled patient-centered health systems (5, 6).

Even though COVID-19 has caused massive problems in the healthcare system, it has forced the majority of countries to bridge the DHI gap (7). Different studies across the globe assessed the potential of DHIs in the fight against COVID-19, including their role in service delivery, health literacy, disease surveillance, treatment and vaccination, and program follow-up (7–10). At the start of the pandemic, innovative digital health-based analysis of social media data and news reports assisted in forecasting the spread of the disease. Social media platforms and features open up new avenues for educating people including hard-to-reach, recruiting participants in therapeutic trials, and remote-delivering of healthcare. However, there are several digital health implementation challenges and opportunities across countries and territories that need to be compiled to inform policy and practice as the disease is not yet over. This perspective paper argues how digital health interventions assist in the fight against COVID-19 and their opportunities, implications, and limitations.

### Opportunities for implementing digital health interventions in the era of COVID-19

The COVID-19 pandemic served as a veritable testing ground for emerging digital health concepts and practices. DHIs provide enormous support in the social distancing time that interrupted healthcare service delivery. The application of telemedicine has facilitated service continuity with great potential to protect both patients and care providers (11). Hospitals' closure forced the public to seek and practice alternative digital health solutions such as smartphones to connect with their clinicians and follow-up routine care (8, 12). The use of digital health for COVID-19 screening reduced the number of visits to emergency departments while also improving healthcare system organization (13). mHealth, telemedicine, eHealth, and a variety of other mobile applications rose to prominence during lockdowns and were widely used for diagnosis, clinical care, and patient follow-up, demonstrating their potential beyond serving marginalized and underprivileged communities (14).

Virtual communication platforms facilitated remote interactions between healthcare professionals and patients, as well as the creation of operational management dashboards for optimizing workflows, resources, and patient-centered care. Several healthcare institutions have been drawn to cloud technologies in order to implement discrete COVID-19-related functionality such as testing, diagnostics, monitoring, triage, and patient consultations. large numbers of research papers accessible through the COVID-19 Open Research Database can be analyzed quickly using machine learning to extract relevant knowledge about drugs that might be beneficial for the treatment of COVID-19. By generating data summaries from multiple sources, artificial intelligence platforms enabled real-time monitoring of patients in high-risk settings for COVID-19. Insurance companies use of health-tracking reward programs that encourage the application of wearable health technologies, though implementation has been straightforward.

We have included case studies from three different countries to demonstrate how DHI has been used to support the healthcare system during the pandemic.

### Case 1: China

In response to the pandemic, a hospital in China's Guangdong province that is well-known for its smart services used an existing platform to launch COVID 19 responsive services. These included information hubs, e-Consultation and screening, remote symptom monitoring, and psychological support. The system was web-based and linked users from social media sites (WeChat Facebook/Twitter equivalent), a phone App, and a website. The hospital reported that they saw a drastic drop in outpatient visits during the pandemic lockdowns but recorded high usage of the online services even at the height of the pandemic. This implies that more people had been restricted access to physical health services but they still got the healthcare services through DHIs. They also reported that the system allowed them to better triage patients who needed emergency response by providing remote care and hence, reducing the workload on clinicians and encouraging social distancing (15).

### Case 2: Kenya

A provider-to-provider (P2P) asynchronous telemedicine model developed and implemented during the second year of COVID-19 in Kenya facilitated the delivery of essential health services (16). Since 2011, the Addis Clinic telemedicine platform has been providing access to specialized medical experts for frontline healthcare providers treating patients in low-resource settings. Healthcare providers heavily used this digital health platform in Kenya during the outbreak, indicating they found it very useful to them during the outbreak. Despite some of the infrastructure and network connectivity challenges present in the country, the provider-to-provider telemedicine platform was a viable option for receiving clinical recommendations from medical experts located remotely and sustaining essential health services.

### Case 3: Ethiopia

A COVID-19 e-health educational intervention in Ethiopia targeting healthcare workers delivered a series of three 1-h medical seminars on COVID-19 prevention and treatment. The study collected post-seminar evaluation data from the participants using a questionnaire. The findings demonstrated the promising potential of transitioning healthcare training and delivery from an in-person to a digital medium in low-resource settings like Ethiopia (17). COVID-19 had a significant burden on patients, healthcare providers, and the healthcare system in general in Ethiopia, where the Ethiopian government and its partners intervened to sustain healthcare services (18–20).

### Case 4: Vietnam

Since the beginning of the COVID-19 pandemic, seven major digital applications have been implemented in Vietnam (21). They have been classified into four categories based on their main purpose, including surveillance and contact tracing, health communication, telemedicine, and Artificial Intelligence to support diagnosis and treatment. The eCDS software was primarily focused on reporting case-based hospital admissions *via* an electronic system. Furthermore, two mobile apps (NCOVI and Vietnam Health Declaration) were created to record electronic health declaration forms for domestic and international travelers for case monitoring and surveillance (21).

## Challenges and limitations to implementing digital health technologies

Despite a history of public health crises, data-sharing agreements and transactional standards do not exist uniformly between institutions, impeding a foundational infrastructure to meet data-sharing and integration needs for public health advancement. COVID-19 has revealed not only the need for data sharing but also the importance of serious evaluation and ethical considerations in the emerging field of digital health (22, 23). One of the main challenges in the biomedical research community and one of the contributors to international information sharing is maintaining control over the constantly generated data while simultaneously promoting their active use for generating scientific discoveries. Obtaining informed consent has been also a significant challenge in providing transparency about the kind of documents collected and which third parties able to access patient's data. Procedures were carried out remotely and/or *via* electronic consent during the pandemic; however, not all healthcare facilities were prepared to provide digital consent, prompting some scientists to create their own way of acquiring consent and electronic signature for participants in therapeutic trials. Issues such as potential participants' access to technology and an absence of user-friendly functionalities to interpret consent documents exacerbated the preceding problem of having complicated, lengthy, and technical consent documents in studies that used e-consent platforms.

There is a desperate need for strengthening resource capacity for effective implementation and evaluation of digital health technologies, taking into account the needs and priorities of countries (24–26). Inequalities in infrastructure and access to the internet and electricity are among the major challenges in the implementation and scale-up of DHIs in resource-limited settings. Digital health technologies were recommended to help patients adhere to their treatment; however, for optimal implementation of such technologies, trials evaluating the effectiveness of remote treatment are critically needed (19, 27–29).

Digital health literacy in the general population often determines the acceptability and adaptability of digital health solutions in a given country and this has been witnessed in the era of COVID-19. Low levels of digital health literacy were found to have a significant association with an individual's COVID-19 precautionary practices, information accuracy, vaccine hesitancy, and subjective wellbeing (30–34). The lack of qualified and skilled professionals in digital health is one of the main barriers to digital health applications, especially in resource-constrained settings (35).

In many resource-limited countries, the lack of policies and strategies, governance structures standard operating procedures, and financial resources hamper the successful deployment and implementation of DHIs (35). Sufficient and sustainable financial mechanisms are not in place for large-scale deployment of approved DHIs. Significant number of such technologies are not culturally adapted to incorporate the local context and facilitate easy understanding by patients and providers (36). The move away from traditional face-to-face care and treatment to digital health-enabled remote care and monitoring was not straightforward and the pros and cons vary by country, program, and the type of digital health technology employed (37).

## Discussion

During the COVID-19 pandemic, there were a large number of patients seeking healthcare, putting a significant strain on healthcare providers. As a result, remote patient monitoring and the use of mHealth applications became an essential part of healthcare delivery. Telemedicine for remote consultation and the use of health devices such as pulse oximetry was a pivotal DHI in the COVID-19 pandemic. As mentioned in the case studies, applications designed for patient surveillance and contact tracing were crucial in reducing the pandemic's impact. The pandemic brought all stakeholders' priorities in various aspects of digital health adoption in line, including expedited regulatory approval of clinical studies. However, the widespread use of DHIs has been hampered by a lack of infrastructure, equitable access to the internet and electricity, and evidence-based digital health standards and data governance systems.

In the COVID-19 era, electronic medical records provide large amounts of data that can be used to generate research evidence, but these data require quality assurance and valid sampling procedures. Such disadvantages reduce the quality of research driven by data captured through digital health. Privacy is a concern in the digitalization of health care, where data generated by digital health must be safeguarded. It is also important to clearly explain to all stakeholders how their data will be used and protected.

DHI implementation during COVID-19 faces some unique challenges in resource-constrained settings. There were reservations and uncertainties regarding the use, adaptation, and integration of DHIs into the wider scope healthcare system before COVID-19, which slowed information dissemination and prompt responses. In these settings, insufficient financial resources, electricity supply, internet connectivity, and a trained workforce impede DHI implementation during COVID-19.

Because of their diverse nature and types, the lack of homogeneous interventions creates a major obstacle in implementing DH interventions. Such distinctions may limit model generalization and understanding of DH effectiveness (13, 78). During the pandemic, most low- and middle-income countries faced serious issues with the practicability and desirability of digital technology by care providers. This is due to a shortage of training on new technology tools, insufficient technical assistance, internet connectivity problems, and also other administrative complexities. Thus, care providers in those countries find it difficult to adopt and use digital health solutions in the healthcare system. Acknowledging that many patients need recommendations or guidance, the use of digital technologies in providing guidance services has already been previously deployed in some countries and can be adopted as an alternative to physical visits in hospitals.

We can conclude from the preceding discussion that DHIs have enabled countries to mitigate the impact of the COVID-19 pandemic, paving the way for and reshaping the next generation of healthcare. COVID-19 has aided in the massive deployment of DHIs for immediate outbreak response. To better move the DHIs momentum forward and sustainably mitigate COVID-19, equitable access to approved digital health technologies, political commitment, collaboration, stakeholder cooperation, and workforce capacity building are required. Beyond COVID-19, there may be an opportunity to set an additional focus and implement policies throughout the health system to support the potential use of DHI solutions.

The implication of this paper for policy and practice is summarized in Table 1.

**Table 1:** Implications of the paper for digital health interventions policy and practice in the era of COVID-19.

**Current situation and problems**	**Suggested solutions**
Lack of uniformity in data sharing among institutions	Creating standardized system
Unable to take proper consent during the pandemic	Preparing a digital signature for taking a remote consent
Difficulty using digital health technologies by healthcare providers and patients	Giving proper training
Poor infrastructure for DHI implementation	Political and stakeholders' commitment
Issues of confidentiality and privacy	Establishing standards for data governance systems and anonymization of the identification of the person
Lack of evidence and supporting strategies that describe in detail how DHIs can influence health outcomes, health system efficiencies, and service delivery cost-effectiveness	Conducting evidence-based researches

## Data availability statement

Publicly available datasets were analyzed in this study. This data can be found here: https://osf.io/gvm2z/.

## Author contributions

Study conception, data acquisition, synthesis, and first draft of the manuscript: EG and TA. Data acquisition and synthesis: SM, CW, VK, HT, LC, JA, BS, SS, MA, and TM. Resource acquisition and critical review of the draft manuscript: TM. All authors reviewed and approved the final version for publication.
